# Cone Beam Computed Tomography in the Assessment of the Effectiveness of Physical Therapy with the Use of the Electromagnetic Field Combined with Light Radiation Emitted by LEDs in the Treatment of Inflammation of the Paranasal Sinuses—A Case Study

**DOI:** 10.3390/ijerph192013570

**Published:** 2022-10-20

**Authors:** Konrad Kijak, Grzegorz Cieślar, Małgorzata Kowacka, Piotr Skomro, Helena Gronwald, Adam Garstka, Danuta Lietz-Kijak

**Affiliations:** 1 Student Scientific Society, Department of Internal Medicine, Angiology and Physical Medicine, Medical University of Silesia in Katowice, 40-055 Katowice, Poland; 2 Department of Internal Medicine, Angiology and Physical Medicine, Medical University of Silesia in Katowice, 40-055 Katowice, Poland; 3 Provincial Specialist Hospital No. 4 in Bytom, 41-902 Bytom, Poland; 4 Department of Propaedeutic, Physical Diagnostics and Dental Physiotherapy, Faculty of Medicine and Dentistry, Pomeranian Medical University, 70-204 Szczecin, Poland

**Keywords:** volumetric tomography, cone beam computed tomography (CBCT), Extremely Low Frequency-Electromagnetic Field (ELF-EMF), LED light therapy, sinus diseases

## Abstract

Modern high-resolution volumetric tomography, commonly known as cone beam computed tomography (CBCT), is one of the most innovative imaging techniques which can provide views of anatomical structures not attainable by conventional techniques. Magnetic field LED therapy is a physical therapy method, combining the effects of the Extremely Low Frequency-Electromagnetic Field (ELF-EMF) and high-power light radiation emitted by Light Emitting Diodes (LEDs). The method has been widely applied in the treatment and rehabilitation of complications of many medical conditions, including in dentistry. The aim of this study was to use CBCT to assess the effectiveness of the simultaneous use of electromagnetic field and LED light in the physical therapy of paranasal sinusitis. Treatments employing the electromagnetic field combined with LED light were administered to a 39-year-old female outpatient of the physiotherapy ward for rehabilitation therapy of paranasal sinusitis. Normal sinus pneumatization was restored almost completely. Reduction in the swelling of the sinus mucosa was so significant that even the pneumatization of the ethmoid bulla was restored. Physical therapy with the simultaneous use of ELF-EMF and LED light was found to be effective in the rehabilitation of the patient with paranasal sinusitis. Positive effects of the treatment were confirmed by CBCT findings.

## 1. Introduction

Different X-ray imaging techniques used in dentistry differ in terms of accuracy in the reproduction of anatomical details of the diagnosed area [1,2,3]. Cone beam computed tomography (CBCT) imaging has an instrumental advantage in that it can be used to reconstruct a three-dimensional image, providing a detailed insight into the topography of skeletal structures in a chosen projection [3,4,5,6]. CBCT is also successfully used in otolaryngology. Due to the accuracy of this imaging technique, it is possible to simultaneously diagnose and plan treatment for paranasal sinusitis. Importantly, this technique is safe for the patient since the dose of ionizing radiation in CBCT is reduced, about 20–30 times lower than the conventional multi-slice CT. The average dose of radiation in CBCT is in the range of 20–650 µSv, depending on the size of the field of view and image resolution [7,8,9,10]. In turn, magnetic field LED therapy is a popular physical therapy modality with a high therapeutic efficacy, due to the combined effects of Extremely Low Frequency-Electromagnetic Field (ELF-EMF) and high-power light emitted by Light Emitting Diodes (LEDs), producing red light (R) (wavelength 630 nm) and infrared light (IR) (wavelength 855 nm) radiation [11,12,13,14,15]. The electromagnetic field used in this method usually has magnetic flux density of 15 μT, and frequency in the range of 180–195 Hz [16,17]. In dental applications, it has been shown to provide regenerative effects and nerve stimulation. The method can be used in the treatment of large inflammatory lesions of periapical tissues, soft tissue cysts, dry socket, neuralgia, lingual nerve regeneration, tooth replantation sites and mandibular fractures. The electromagnetic field combined with high-power LED light activates calcium channels, transmembrane ion transport, and stimulates enzyme activity. Regenerative effects primarily come from the intensification of oxygen supply and tissue respiration associated with increased diffusion and oxygen uptake by hemoglobin and cytochromes. Increased oxygen uptake stimulates tissue respiration, DNA synthesis, and accelerates the mitotic cycle. The anti-inflammatory effect is associated with the upregulation of c-AMP and prostaglandin E synthesis, which promotes the accumulation of c-AMP and reduces the secretion of inflammatory mediators from neutrophils, basophils, mast cells and lymphocytes [16,17,18,19,20,21,22]. In turn, the analgesic effect of this method can be attributed to the reduction of nerve impulse conduction in afferent nerve fibers due to the hyperpolarization of neuron membranes, as well as to the increase in synthesis and secretion of endogenous opiates (beta-endorphins) in the central nervous system, consequently raising the pain threshold [23,24,25]. Magnetic field LED therapy has found many applications in the treatment and rehabilitation of complications of many medical conditions [26,27,28,29,30,31], including in dentistry [32].

## 2. Aim of Study

The aim of this study was to use cone beam computed tomography with a large field of view to assess the effectiveness of the simultaneous use of electromagnetic field and LED light in the physical therapy of paranasal sinusitis.

## 3. Material and Methods

A 39-year-old female, A.F., presented to the outpatient clinic with recurrent upper respiratory tract infections, which had been treated for 2.5 years with antibiotics and steroids. Periodically, the patient was prescribed an intranasal glucocorticoid—Mometasone nasal spray, to be administered once daily, at 200 µg (two actuations) of the drug into each nostril, for no longer than 3 weeks. Concurrently, the patient took an antibiotic, amoxicillin (750 mg) with clavulanic acid (125 mg), twice daily for no longer than 2 weeks. During the treatment, the patient also rinsed her nose with a saline solution. The main symptoms reported by the patient were: a congested nose making it difficult to breathe, frequent nasal discharge, facial pain, a feeling of swelling, tenderness and discomfort around the eyes, forehead and cheeks. Olfactory loss was also observed. These symptoms had persisted for more than 2 weeks. Pharmacological treatment was unsuccessful, and the patient was offered an otolaryngological surgical intervention. The patient did not consent to the surgery. After ruling out contraindications, the patient was qualified for the study. The patient did not use any pharmacological agents during physical therapy treatments.

To make an accurate diagnosis and establish the etiology of the patient’s symptoms, we used cone beam computed tomography (CBCT), which enabled us to examine both hard tissue structures and soft elements of the mucous membrane in the paranasal sinuses. Tomography also ruled out odontogenic sinusitis. Volumetric tomographic imaging of the facial skeleton was performed using an i-CAT Next Generation (ISI) system with a wide field of view (FOV) (size 17 cm × 23 cm). Based on personal observation of selected cases, we analyzed the suitability and effectiveness of this radiographic technique for the diagnosis of pathological lesions in the mucous membrane of maxillary sinuses. The patient was examined in a sitting position with a stabilized head to avoid motion-induced artefacts, and Quantum IQ software was used to acquire high-quality images of soft tissues. All scans were taken in the same radiography laboratory using the same apparatus and exposure parameters: 110 kV, 5 mA, time 17 s and −0.3 voxel resolution.

**Figure 1:**
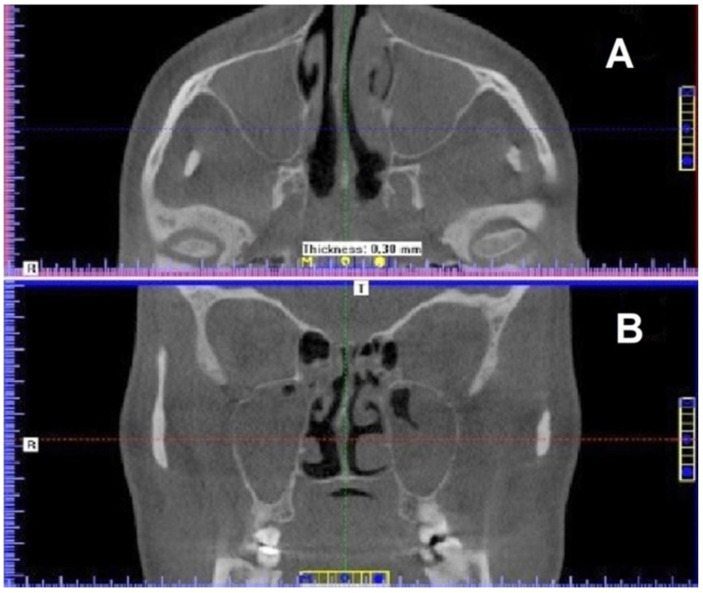
Maxillary sinuses visualized as dark (low-density) areas. (**A**) axial view; (**B**) coronal view (CBCT scans).

Volumetric tomographic imaging revealed significantly decreased aeration of maxillary sinuses (Figure 1).

**Figure 2:**
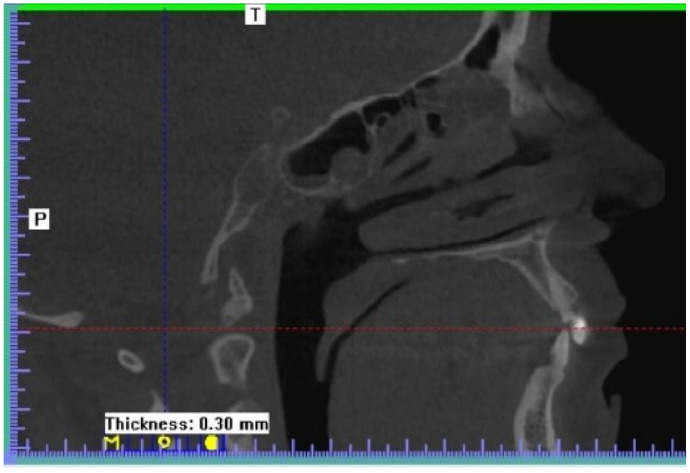
Mucosal thickening in ethmoid cells and at the bottom of the sphenoid sinus—CBCT scan—sagittal view.

CBCT also showed mucosal thickening in ethmoid cells and at the bottom of the sphenoid sinus (Figure 2).

**Figure 3:**
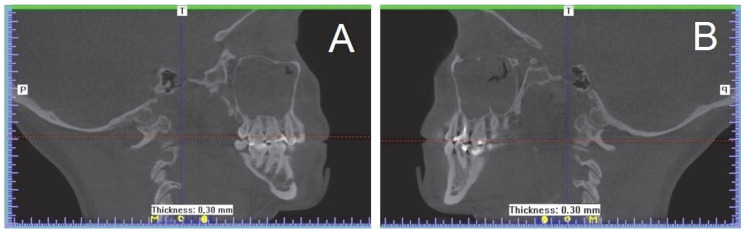
Maxillary sinuses in sagittal view: (**A**) Right and (**B**) left. (CBCT scans).

A detailed analysis of the images made it possible to rule out the dental origin of pathological lesions (Figure 3).

**Figure 4:**
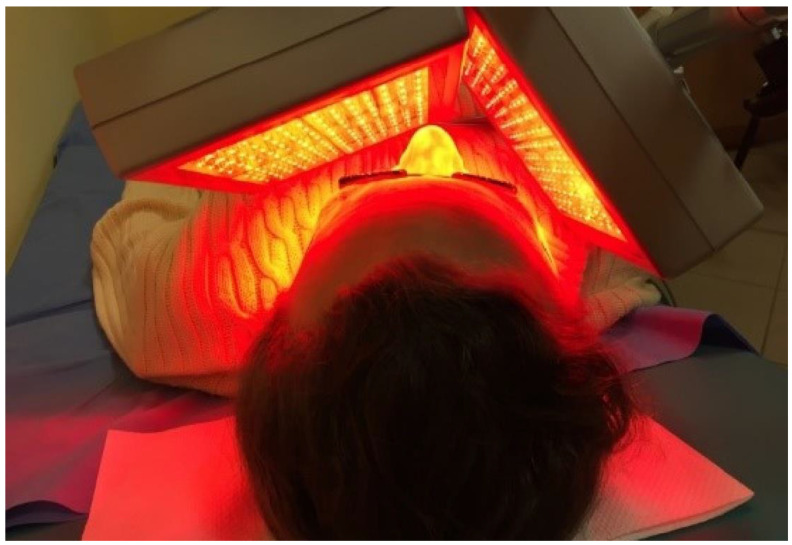
Therapeutic treatment with the simultaneous use of electromagnetic field and high-power light emitted by LEDs, applied by means of two panel applicators of the Viofor JPS Magnetic Light device.

The Viofor JPS System (Med and Life, Komorów, Poland) was used in the treatment under discussion. The system features a control unit and proprietary applicators for low frequency magnetic therapy and combination therapy, namely Pulsed Magnetic Field Therapy (Magnetotherapy), Pulsed Magnetic Field Stimulation (Magnetostimulation), and Pulsed Magnetic Field LED Therapy. With the wide range of applicators, it is possible to perform topical treatments on small and mid-sized surfaces, as well as whole-body treatments. The patient underwent a series of physical therapy treatments, with 30 daily sessions lasting 20 min each, and each using two panel applicators of the VIOFOR JPS Magnetic Light device containing 560 LEDs emitting mixed radiation: red light (wavelength 630 nm, power 500 mW) and infrared light (wavelength 855 nm, power 3400 mW), with a frequency of light impulses of 181.8 Hz. This generated a simultaneous electromagnetic field with magnetic flux density of 15 μT and magnetic field frequency in the range of 180–195 Hz (Figure 4).

The effectiveness of the physical therapy was assessed by comparing the CBCT images acquired before and after the series of treatments, with a focus on the condition of the paranasal sinuses. The contraindications for this therapy included: pregnancy and breastfeeding, acute inflammation, skin cancer, autoimmune diseases, light sensitivity, epilepsy, treatment with vitamin A derivatives, photophobia, porphyria, use of medications prone to photodegradation, tuberculosis, (uncontrolled) diabetes, severe viral, bacterial and fungal infections and electronic implants. During the procedure, the patient wore protective glasses.

## 4. Results

**Figure 5:**
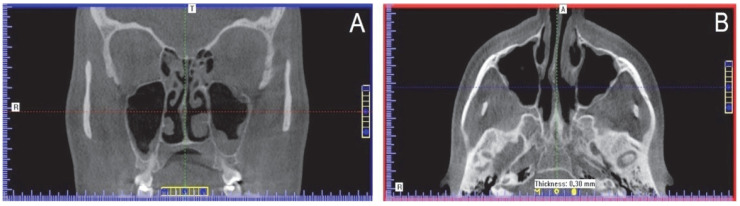
Image of maxillary sinuses after 30 sessions of electromagnetic field and LED light therapy. (**A**) coronal view; (**B**) axial view. (CBCT scans).

Follow-up imaging was performed at the end of the 30-day therapy. CBCT scans revealed a marked regression of inflammation in all paranasal sinuses, which was correlated with improved patient comfort. Normal sinus pneumatization was restored almost completely (Figure 5).

**Figure 6:**
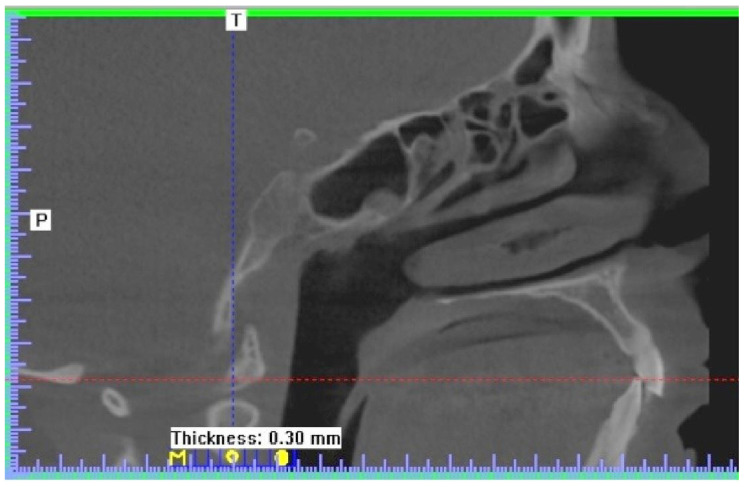
Ethmoid bulla and the sphenoid sinus—status after a series of 30 physical therapy sessions. (CBCT scan).

Reduction in the swelling of the sinus mucosa was so significant that even ethmoid bulla pneumatization was restored. Pathological lesions in the sphenoid sinus also resolved completely (Figure 6). At the bottom of the maxillary sinuses, mucosal thickening was still visible. In the right maxillary sinus, the maximum mucosal thickness was 3–4 mm. In the left maxillary sinus, the maximum mucosal thickness was 3–7 mm. The patient follow-up is continuing. She will require a second round of physical therapy. After the series of treatments discussed in this paper, the patient reported manifest improvement, including elimination of frontal headaches, eye and nose pain, nasal discharge and congestion.

## 5. Discussion

Sinusitis is a broad clinical syndrome with the common feature of mucosal inflammation. It is a complex of symptoms rather than a specific disease [33,34]. Objective evidence of chronic rhinosinusitis can be obtained by physical examination (anterior rhinoscopy and endoscopy) or radiography, preferably computed tomography of the sinuses [35,36]. The primary methods of treatment are pharmacological, using corticosteroids and antibiotics. First line pharmacological treatment mainly consists of topical intranasal and oral corticosteroids. Corticosteroids, which have very broad anti-inflammatory properties, also have the strongest evidence for efficacy. If the treatment fails, surgical options are used. Antibiotics are likely to be effective in a subpopulation of patients, but the different phenotypes and endotypes that make up sinusitis have been poorly defined to date. For some phenotypes, early surgery, as well as biologic therapy, may be more effective and cost-effective [33,34,37]. With intranasal corticosteroids, caution is advised, especially in children, pregnant women and elderly patients, and even more so in patients with comorbidities such as asthma, whose total steroid intake may be high due to the administration of both intranasal and inhaled corticosteroids [37]. In the present case study, physical therapy using LED light and electromagnetic field produced very good therapeutic results. This method does not have the limitations of corticosteroids and is not invasive like surgical procedures. The two physical factors have been observed to counteract inflammation by providing regenerative, analgesic, anti-inflammatory and antiedematous, as well as antimicrobial, angiogenetic and vasodilatory effects. There are reports of an improvement in tissue oxygen utilization and favorable modification of the immune function and coagulation system [38,39,40,41,42,43]. The above is a case report, and so the authors cannot explain why mucosal thickening in certain areas of the paranasal sinuses was reduced to a greater extent. Perhaps this is related to the different volume of the sinuses. Even after volume restoration of the maxillary sinuses, mucosa thickening was still seen.

## 6. Conclusions

Cone beam computed tomography can identify the most inconspicuous pathological changes in the paranasal sinuses and help exclude the dental origin of sinus inflammation.

Physiotherapy with the simultaneous use of ELF-EMF and LEDs light proved to be effective in the rehabilitation of a sinusitis patient. High therapeutic efficacy, lack of significant side effects and good tolerance of the treatment make this therapy potentially useful as a valuable adjunct to conventional symptomatic treatment. The obtained results will encourage further studies in larger patient samples and with longer therapy and follow-up periods.

## Abbreviations

CBCT	Cone Beam Computed Tomography
ELF-EMF	Extremely Low Frequency—Electromagnetic Field
LED	Light Emitting Diode
IR	infrared light
R	red light
